# Low-Complexity Rate-Distortion Optimization of Sampling Rate and Bit-Depth for Compressed Sensing of Images

**DOI:** 10.3390/e22010125

**Published:** 2020-01-20

**Authors:** Qunlin Chen, Derong Chen, Jiulu Gong, Jie Ruan

**Affiliations:** School of Mechatronical Engineering, Beijing Institute of Technology, Beijing 100081, China; 3120170115@bit.edu.cn (Q.C.); cdrmy@263.net (D.C.); 2220175016@bit.edu.cn (J.R.)

**Keywords:** compressive sensing, CS acquisition, quantization, rate-distortion optimization, bit-rate model, relative PSNR model, image processing

## Abstract

Compressed sensing (CS) offers a framework for image acquisition, which has excellent potential in image sampling and compression applications due to the sub-Nyquist sampling rate and low complexity. In engineering practices, the resulting CS samples are quantized by finite bits for transmission. In circumstances where the bit budget for image transmission is constrained, knowing how to choose the sampling rate and the number of bits per measurement (bit-depth) is essential for the quality of CS reconstruction. In this paper, we first present a bit-rate model that considers the compression performance of CS, quantification, and entropy coder. The bit-rate model reveals the relationship between bit rate, sampling rate, and bit-depth. Then, we propose a relative peak signal-to-noise ratio (PSNR) model for evaluating distortion, which reveals the relationship between relative PSNR, sampling rate, and bit-depth. Finally, the optimal sampling rate and bit-depth are determined based on the rate-distortion (RD) criteria with the bit-rate model and the relative PSNR model. The experimental results show that the actual bit rate obtained by the optimized sampling rate and bit-depth is very close to the target bit rate. Compared with the traditional CS coding method with a fixed sampling rate, the proposed method provides better rate-distortion performance, and the additional calculation amount amounts to less than 1%.

## 1. Introduction

Compressed sensing (CS), also known as compressive sensing or compressive sampling, shows that a small group of linear, non-adaptive measurements can reconstruct finite-dimensional signals with sparse or compressible representations [1,2,3,4,5,6]. By simultaneous sampling and data compression, a CS-based imaging system abandons the traditional architecture so that the encoder does not require too much time and hardware [7,8,9,10].

Many compressive sensing studies describe the constraints of the measurement budget, such as allocating sensing resources for regions of interest [11,12] and adaptive sampling for block compressive sensing [13,14]. However, real-valued CS measurements must be quantified in CS-based imaging systems, and there is a given bit-budget constraint rather than a measurement budget.

In a classical imaging system, the quantizer determines the bit rate and the distortion [15]. However, compressive sensing is different from that situation. The bit rate and distortion are determined by the sampling rate and bit-depth in the CS-based imaging system. Therefore, there is a tradeoff between the sampling rate and the bit-depth in CS-based imaging systems with bit-budget constraints [16].

In order to obtain a high-quality image, rate-distortion optimization (RDO) must be performed on the encoder to allocate the optimal sampling rate and bit-depth to minimize the distortion of the reconstructed image. As far as we know, there are few research results on the joint optimization of sampling rate and bit-depth. Liu et al. [17] proposed a distortion model of compressive video sampling for optimizing sampling rate and bit-depth, but the parameters of the model were closely related to the video sequence. Jiang and Yang [18] proposed an improved Lagrange multiplier method to optimize the number of measurements and quantization step size, but the method did not consider the computational complexity of rate-distortion cost, which is not conducive to practical applications. Some problems occur when calculating the bit rate of the CS encoder. In [14], the bit rate was calculated directly by the sampling rate and the bit-depth. The effect of entropy coding was ignored, which reduces the utilization of the target bit rate. Although entropy coding was introduced in [13], there was more computational complexity for the encoder because the bit-rate cost was calculated through the actual coding results.

Due to the low computational complexity of CS, the computational complexity of rate-distortion optimization cannot be too high at the encoder. In this paper, we introduce a uniform scalar quantization and entropy coder to the CS, developing a CS-based imaging framework with RDO wherein the sampling rate and bit-depth are jointly optimized. One of the main contributions of this paper is the bit-rate model, which reveals the relationship between bit rate, sampling rate, bit-depth, and characteristics of partial measurements. Another contribution is to introduce a relative peak signal-to-noise ratio (PSNR) model and use a feedforward neural network to teach the relative PSNR model to estimate distortion. Finally, a method of optimizing sampling rate and bit-depth is proposed by using the bit rate and the relative PSNR model.

The rest of this paper is organized as follows. Section 2 introduces the rate-distortion optimization problem for the parameters of the CS-based imaging system, which are the sampling rate and the bit-depth. The proposed bit-rate model and relative peak signal-to-noise ratio (PSNR) model are discussed in more detail in Section 3 and Section 4, respectively. Section 5 describes and discusses the rate-distortion optimization method for the sampling rate and bit-depth with the bit-rate model and the relative PSNR model. Section 6 describes the experiments and results, and we draw some conclusions in Section 7.

## 2. Problem Formulation

Let $x\in {\mathbb{R}}^{N\times 1}$ represent the vector form of the image after raster scanning. Assume $\theta \in {\mathbb{R}}^{N\times 1}$ is the coefficient of x in the orthogonal transform $\Psi \in {\mathbb{R}}^{N\times N}$, that is x=Ψθ. When x can be approximately represented by only K of N non-zero coefficients, x is called a K-sparse signal. Natural images are usually sparse in discrete cosine transform (DCT) and discrete wavelet transforms [19,20]. The CS theory states that the sparse signal x can be accurately reconstructed through M(M<N) linear and non-adaptive measurements with an overwhelming probability. The measurement vector $y\in {\mathbb{R}}^{M\times 1}$ is obtained by the following:

(1) y=Φx,

where $\Phi \in {\mathbb{R}}^{M\times N}$ is the measurement matrix which should satisfy restricted isometry property (RIP) and incoherence property [1,6,21], and a Gaussian random matrix is often used [5]. When reconstructing the image signal x from the measurement vector y, y=Φx is an ill-posed problem with infinite solutions. In order to obtain a unique solution, the sparsity [1,5] of images is usually used as the prior condition to constrain the solution space of y=Φx. Some other prior conditions exist, such as total variational (TV) minimization [22] and non-local similarity [23], which are also considered as sparsity of an image in a particular transform domain. Moreover, a usual requirement is that the number of measurements meet at least M=O(Klog(N)) to ensure high-quality reconstructed images. $m=\;\frac{M}{N}$ is often called the measurement rate or sampling rate.

In practice, real-valued CS measurements must be mapped to discrete bits by a quantizer. Therefore, the CS acquisition model with quantization [16] is as follows:

(2) $$y_{Q}=Q_{b}(y)=Q_{b}(\Phi x),$$

where $Q_{b}:\mathbb{R}\to \mathbb{Q}$ is a scalar quantization function of b-bit that maps real-valued measurements to discrete sets ℚ with $\left|\mathbb{Q}\right|=2^{b}$. Considering the low complexity requirements of CS encoders, the uniform scalar quantization method is often used [17,18].

In order to improve the compression performance, entropy coding is performed after the quantizer [18]. The encoded data is as follows:

(3) $$c_{enc}=f_{enc}\left(y_{Q}\right),$$

where $f_{enc}:\mathbb{Q}\to \mathbb{C}$ is the encoding function that maps the quantized measurements to the binary codeword $c_{enc}$; arithmetic coding is used in this paper.

After the image is compressed by CS measurement, quantizer, and entropy coder, the average number of bits per pixel (bpp) in the image can be expressed as follows:

(4) $$R=m\cdot L_{y_{Q}},$$

where R is called bit rate and $L_{y_{Q}}$ represents the average codeword length after entropy coding of $y_{Q}$.

In practice, we are often constrained by a bit budget when transmitting or storing the compressed data. At this point, the sampling rate and bit-depth must be balanced [16]. On the one hand, we can increase the depth of the quantization bits by reducing the sampling rate, thereby improving the reconstruction quality. On the other hand, when we reduce the sampling rate, the reconstruction quality will decrease. How to allocate the sampling rate m and the quantization bit-depth b is expressed as an optimization problem based on the rate-distortion criterion and is given as the following:

(5) $$\begin{array}{l} \underset{m,b}{\min}D(x,m,b)\\ s.t.\;R(x,m,b)\leq R_{\max} \end{array},$$

where D(x,m,b) represents the distortion for the image x with sampling rate m and bit-depth b, R(x,m,b) represents the bit rate for the image x with sampling rate m and bit-depth b, and $R_{\max}$ represents the budget for the bit rate of the image compression data.

**Figure 1:**
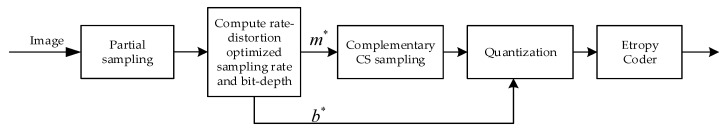
Proposed adaptive compressive sampling framework with rate-distortion optimization.

Compressed sensing can significantly reduce the complexity of the encoder. When solving model (5), the computational complexity is very important for CS-based imaging systems. If the complexity of rate-distortion optimization is too high, this will run counter to our original intention of using CS coding. Because the calculation of the bit rate and distortion far exceeds the calculation of CS acquisition, we proposed a bit-rate model estimating R(x,m,b) and a relative PSNR model estimating distortion. Based on the sampling method in the adaptive compression video sampling framework of [17], we designed a CS-based image coding framework, as shown in Figure 1. The proposed CS framework contains two measurement processes. The first one is a partial measurement whose purpose is to extract the image features for the bit-rate model and relative PSNR model by using a small number of measurements. The second one is complementary CS measurement, which completes CS measurement according to the sampling rate obtained by the rate-distortion optimization.

## 3. Bit-Rate Model

According to Equation (4), the average codeword length $L_{y_{Q}}$ of the entropy coder is the key to calculating the bit rate. The average codeword length can be approximated by information entropy [24]. However, the calculation of information entropy requires the use of all measured value information, which cannot be achieved before the sampling rate is determined. In order to calculate the information entropy of the measurements sampled by a sampling rate m, we proposed estimating the information entropy based on a small number of measured values from the first sampling. However, the information entropy is only the lower boundary of the average codeword length for entropy coding, and there are some errors between the real information entropy and the information entropy estimated by a few measurements. Therefore, we used the second-order Taylor expansion method to approximate the estimation model of information entropy, in which the model can be expressed as the additive model of each characteristic variable. Then, we modified the coefficients of the additive model by fitting off-line data, which can improve the estimation accuracy of the average codeword length $L_{y_{Q}}$.

### 3.1. Estimation of Information Entropy

When sampling with the Gaussian random matrix, the CS measurements obey the Gaussian distribution [25]. Moreover, the density function of the quantized CS measurements follows the distribution of the corresponding real-value CS measurements [26], that is, the quantized measurements also obey the Gaussian distribution, so the information entropy [27] of the quantized CS measurements can be estimated as follows:

(6) $$H_{y_{Q}}\approx \frac{1}{2}\log_{2}\left(2\pi eV_{0}\right),$$

where $V_{0}$ is the variance of the quantized CS measurements.

In order to facilitate the uniform quantization, the measurements are first scaled to the integer interval corresponding to the quantization bit-depth, and then the rounding operation is used. The uniform quantization function can be expressed as follows:

(7) $$Q(y_{i})=round\left(\frac{y_{i}-y_{\min}}{\left(y_{\max}-y_{\min}\right)}(2^{b}-1)\right),$$

where $y_{i}$ is a measurement, $y_{\max}$ is the maximum element of the measurement vector y, and $y_{\min}$ is the minimum element of the measurement vector y. We used the independent random variables ε that obey uniformly distributed U[−0.5,0.5] to represent the rounding error in the uniform quantization function [28], then $Q(y_{i})\approx \frac{y_{i}-y_{\min}}{\left(y_{\max}-y_{\min}\right)}(2^{b}-1)+\varepsilon$. Let $\sigma_{m}^{2}$ denote the variance of the measurements when the sampling rate is m and $\sigma_{\varepsilon }^{2}$ denote the variance of the random variable ε, and $\sigma_{\varepsilon }^{2}=\frac{1}{12}$ can be calculated. Assuming that the variable $y_{i}$ obeys a Gaussian distribution with a variance of $\sigma_{m}^{2}$, and the rounding error variable ε is independent of the variable $y_{i}$, the variance of $Q(y_{i})$ can be expressed as follows:

(8) $$V_{0}\approx \frac{{\left(2^{b}-1\right)}^{2}\sigma_{m}^{2}}{{\left(y_{\max}-y_{\min}\right)}^{2}}+\sigma_{\varepsilon }^{2}.$$

Combining Equations (6) and (8), the information entropy $H_{y_{Q}}$ of the b-bit quantized measurements with sampling rate m is the following:

(9) $$H_{y_{Q}}\approx \frac{1}{2}\log_{2}\left(2\pi e\frac{{\left(2^{b}-1\right)}^{2}\sigma_{m}^{2}}{{\left(y_{\max}-y_{\min}\right)}^{2}}+\frac{2\pi e}{12}\right).$$

According to Equation (9), in addition to the bit-depth b, the variance $\sigma_{m}^{2}$, the maximum measurement $y_{\max}$, and the minimum measurement $y_{\min}$ are the keys to calculating $H_{y_{Q}}$. The measurements must be sampled according to the known sampling rate m, which means that the variance $\sigma_{m}^{2}$, maximum measurement $y_{\max}$, and minimum measurement $y_{\min}$ of the measurements cannot be obtained before the sampling rate m is determined. Therefore, we proposed to perform the first sampling to obtain a small number of measurements before the rate-distortion optimization, and then to use this part of the measurements to extract the features that we needed.

In statistical theory, the characteristics of a sample are often used to estimate the characteristics of the population. Since measurements of different sampling rates can be considered as different samples in the population of measurements, there is a close relationship between the characteristics of the different samples. In this paper, the characteristics of the first sampled measurements were used to estimate the characteristic of the measurement with the sampling rate m. We used the maximum measurement ${y^{\prime }}_{\max}$ and the minimum measurement ${y^{\prime }}_{\min}$ obtained by the first sampling to estimate the maximum measurement $y_{\max}$ and the minimum measurement $y_{\min}$ of the sampling rate m. When using the sample to estimate the population variance, there is an unbiased estimate of the variance [29]:

(10) $$s^{2}=\frac{\sum_{i=1}^{Num}{\left(p_{i}-\bar{p}\right)}^{2}}{Num-1},$$

where $p_{i}(i=1,\ldots ,Num)$ is a sample, $\bar{p}$ is the mean of the samples, and Num is the number of samples. According to the definition of variance, we know that $s^{2}=\frac{Num}{Num-1}\sigma^{2}$. Suppose $\sigma_{0}^{2}$ is the variance of the measurement with the sampling rate $m_{0}$ for the first sampling and $\sigma_{m}^{2}$ is the variance of the measurement with the sampling rate m. We used $s^{2}$ of the first sampled measurement to estimate the $s^{2}$ of the measurement with the sampling rate m, that is:

(11) $$\frac{M_{m}}{M_{m}-1}\sigma_{m}^{2}\approx \frac{M_{0}}{M_{0}-1}\sigma_{0}^{2}\;\sigma_{m}^{2}\approx \frac{M_{0}\left(M_{m}-1\right)}{\left(M_{0}-1\right)M_{m}}\sigma_{0}^{2}\approx \left(\frac{Nm_{0}}{\left(Nm_{0}-1\right)}+\frac{-m_{0}}{\left(Nm_{0}-1\right)}\times \frac{1}{m}\right)\sigma_{0}^{2},$$

where $M_{m}=\mathrm{round}(Nm)$ is the number of the measurement with sampling rate m and $M_{0}=\mathrm{round}(Nm_{0})$ is the number of the measurement with sampling rate $m_{0}$. Let $\Delta_{y_{0}}=\left({y^{\prime }}_{\max}-{y^{\prime }}_{\min}\right)$, combined with $H_{y_{Q}}\approx L_{y_{Q}}$ and Equations (6)–(11); we then obtain the following:

(12) $$L_{y_{Q}}\approx \frac{1}{2}\log_{2}\left({\left(2^{b}-1\right)}^{2}\frac{2\pi e\sigma_{0}^{2}\left(\frac{Nm_{0}}{\left(Nm_{0}-1\right)}+\frac{-m_{0}}{\left(Nm_{0}-1\right)}\times \frac{1}{m}\right)}{\Delta_{y_{0}}^{2}}+\frac{2\pi e}{12}\right).$$

### 3.2. Simplified Model of Average Codeword Length $L_{y_{Q}}$

Model (12) takes the variance as well as the maximum and minimum of the measurements obtained by the first sampling as the main features of estimating the average codeword length. However, there is a particular error between the information entropy and the average codeword length of the entropy coder. In this paper, we constructed an additive model of the average codeword length according to model (12) and solved the coefficients of the additive model by the least-squares method. The obtained additive model minimizes the mean squared error (MSE) between the estimated and actual values of the average codeword length, which improves the accuracy of the estimated average codeword length.

In approximating model (12), we performed a second-order Taylor expansion on functional form $\log_{2}(a\prod_{i=1}^{n}x_{i}+C)$ for the variable $x_{i}$, then obtained an addition form as follows:

(13) $$\log_{2}(a\prod_{i=1}^{n}x_{i}+C)\approx \sum_{i=1}^{n}\log_{2}(x_{i})+\sum_{i=1}^{n}c_{i}x_{i}+c_{n+1},$$

where $c_{i}(i=1,\ldots ,n+1)$ is the second-order Taylor coefficient (see Appendix A).

Consider ${\left(2^{b}-1\right)}^{2},\sigma_{0}^{2},\frac{1}{\Delta_{y_{0}}^{2}},\left(\frac{Nm_{0}}{\left(Nm_{0}-1\right)}+\frac{-m_{0}}{\left(Nm_{0}-1\right)}\times \frac{1}{m}\right)$ in model (12) as variables; according to Equation (13), $L_{y_{Q}}$ is approximated as follows:

(14) $$\begin{array}{cc} L_{y_{Q}}\approx & \frac{1}{2}\log_{2}\left({\left(2^{b}-1\right)}^{2}\frac{2\pi e\sigma_{0}^{2}\left(\frac{Nm_{0}}{\left(Nm_{0}-1\right)}+\frac{-m_{0}}{\left(Nm_{0}-1\right)}\times \frac{1}{m}\right)}{\Delta_{y_{0}}^{2}}\right)\\ & +c_{1}{\left(2^{b}-1\right)}^{2}+c_{2}\sigma_{0}^{2}+c_{3}\frac{1}{\Delta_{y_{0}}^{2}}+c_{4}\left(\frac{Nm_{0}}{\left(Nm_{0}-1\right)}+\frac{-m_{0}}{\left(Nm_{0}-1\right)}\times \frac{1}{m}\right)+c_{5} \end{array},$$

where $c_{i}(i=1,\ldots ,5)$ is the Taylor coefficient and can be obtained according to the appendix. The first item in Equation (14) can be expanded as follows:

(15) $$\begin{array}{l} \frac{1}{2}\log_{2}\left({\left(2^{b}-1\right)}^{2}\frac{2\pi e\sigma_{0}^{2}\left(\frac{Nm_{0}}{\left(Nm_{0}-1\right)}+\frac{-m_{0}}{\left(Nm_{0}-1\right)}\times \frac{1}{m}\right)}{\Delta_{y_{0}}^{2}}\right)\\ =\frac{1}{2}\log_{2}\left(\frac{Nm_{0}}{\left(Nm_{0}-1\right)}+\frac{-m_{0}}{\left(Nm_{0}-1\right)}\times \frac{1}{m}\right)+\frac{1}{2}\log_{2}\left({\left(2^{b}-1\right)}^{2}\right)+\frac{1}{2}\log_{2}(\sigma_{0}^{2})-\frac{1}{2}\log_{2}(\Delta_{y_{0}}^{2})+\frac{1}{2}\log_{2}(2\pi e) \end{array}$$

Due to the limited range of parameters, we approximated the logarithmic function in Equation (15) by the square root function, and approximated $\frac{1}{2}\log_{2}\left({\left(2^{b}-1\right)}^{2}\right)=\log_{2}(2^{b}-1)\approx \log_{2}(2^{b})=b$. Combining Equations (12), (14) and (15), we constructed an additive model of average codeword length as follows:

(16) $$L_{y_{Q}}={c^{\prime }}_{1}b+{c^{\prime }}_{2}{\left(2^{b}-1\right)}^{2}+\frac{{c^{\prime }}_{3}}{m}+{c^{\prime }}_{4}\sigma_{0}^{2}+{c^{\prime }}_{5}\Delta_{y_{0}}^{}+\frac{{c^{\prime }}_{6}}{\Delta_{y_{0}}^{2}}+{c^{\prime }}_{7},$$

where ${c^{\prime }}_{1}\sim {c^{\prime }}_{7}$ are model coefficients which are obtained by using the training dataset to fit model (16). Combining Equation (4) with model (16), we obtained the following bit-rate model:

(17) $$R=m\cdot L_{y_{Q}}=m({c^{\prime }}_{1}b+{c^{\prime }}_{2}{\left(2^{b}-1\right)}^{2}+{c^{\prime }}_{4}\sigma_{0}^{2}+{c^{\prime }}_{5}\Delta_{y_{0}}^{}+\frac{{c^{\prime }}_{6}}{\Delta_{y_{0}}^{2}}+{c^{\prime }}_{7})+{c^{\prime }}_{3}.$$

Model (17) has no logarithmic operation, and the maximum ${y^{\prime }}_{\max}$, the minimum ${y^{\prime }}_{\min}$, and the variance $\sigma_{0}^{2}$ of the first sampling can be used to estimate the bit rate at the sampling rate m and bit-depth b, which significantly reduces the computational complexity for estimating the bit rate.

## 4. Relative PSNR Model

As the objective function of the optimization problem (5), distortion is often measured by the error between the original image and the reconstructed image, such as the sum of absolute difference (SAD), mean squared error (MSE), and peak signal-to-noise ratio (PSNR). Due to the complexity of the CS reconstruction algorithm is much higher than the complexity of CS sampling, directly calculating the distortion loses the advantage of low complexity. Therefore, estimating the distortion ensures low complexity for the CS-based encoder. However, in addition to measurement, factors affecting the reconstructed image include the reconstruction algorithm and the degree to which the original image matches the prior constraints. The latter two factors cannot be described objectively, which makes it difficult to directly estimate the error between the original image and the reconstructed image. Since distortion is used to judge the quality of CS coding parameters, the best CS coding parameters can also be solved by the level of distortion. Therefore, we proposed relative peak signal-to-noise ratio (relative PSNR) instead of distortion as the objective function. Relative PSNR is used to measure the difference of PSNR between the reconstructed image and the original image with different parameters in the same image. Although the relative PSNR cannot represent the error between the original image and the reconstructed image, it can be used to evaluate the quality level of the reconstructed image under different parameters. The relative PSNR comes from the PSNR comparison of the same reconstruction algorithm, where we can abandon the impact of the reconstruction algorithm. Thus, the factors that estimate the relative PSNR come mainly from the sampling rate m, the bit-depth b, and the image.

### 4.1. Relative PSNR

The relative PSNR reflects the level of the peak signal-to-noise ratio, which can achieve the same effect as the PSNR for the optimization of sampling rate m and the bit-depth b. The peak signal-to-noise ratio is often used to evaluate the visual quality of the reconstructed image. The relative PSNR not only reflects the level of distortion but also demonstrates the quality of the decoded visual quality. Let $f(b,m,x)=10\times \log_{10}\left(\frac{255^{2}N}{{\left\|x^{\prime }-x\right\|}_{2}^{2}}\right)$ denote the PSNR between the original image x and the image $x^{\prime }$ reconstructed by the measurements obtained by the parameter (b,m). Several relative peak signal-to-noise ratios can be constructed according to f(b,m,x), denoted as F(b,m,x), as in Equations (18)–(21).

(18) $$F_{1}(b,m,x)=f(b,m,x)-f(b_{1},m_{1},x),$$

(19) $$F_{2}(b,m,x)=\frac{f(b,m,x)}{f(b_{1},m_{1},x)},$$

(20) $$F_{3}(b,m,x)=\frac{f(b,m,x)-f(b_{1},m_{1},x)}{f(b_{1},m_{1},x)},$$

(21) $$F_{4}(b,m,x)=\frac{f(b,m,x)-f(b_{1},m_{1},x)}{\left|f(b_{2},m_{2},x)-f(b_{1},m_{1},x)\right|},$$

where $b_{1},m_{1}$ and $b_{2},m_{2}$ are reference parameters for the relative PSNR and are known fixed values. It is easy to prove that the optimization result using $F_{1}、F_{2}、F_{3}、F_{4}$ is consistent with the optimization result using f for the parameters (b,m).

### 4.2. Relative PSNR Model with Feedforward Neural Network Learning

In order to accurately reveal the mapping model between relative PSNR, sampling rate m, and bit-depth b, we used a four-layered feedforward neural network to train the map between relative PSNR and its factors. The four-layered feedforward neural network is not necessary to reveal the mapping relationship between variables in advance, and the backpropagation algorithm is used to learn the mapping between input and output [30,31]. The feedforward neural network can minimize the loss function between the estimated value and the real value, and is widely used in regression prediction [32,33].

The input of the four-layered feedforward neural network is significant for estimating the accuracy of the relative PSNR. The CS image reconstruction model typically consists of measurement data fidelity and sparsity of an image in a particular transform domain. When the sampling rate and the bit-depth are fixed, the reconstruction quality of the image is closely related to the sparsity. According to the large-scale random matrix spectrum analysis theory, the literature [34] infers that the sparsity of the signal can be estimated based on the average energy of the measurements, because the average energy of the measurements can be calculated based on the variance and the mean (Equation (22)). To increase the diversity of the input variables, we used the variance and the mean as an alternative to sparsity as follows:

(22) $$\frac{\sum_{i=1}^{M_{0}}y_{i}^{2}}{M_{0}}=\sigma_{0}^{2}+{\bar{y}}_{0}^{2}.$$

**Figure 2:**
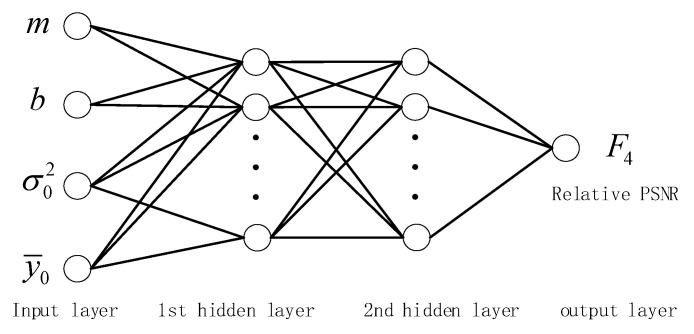
Four-layer feedforward neural network model for the relative peak signal-to-noise ratio (PSNR).

Therefore, we proposed the sampling rate m, the bit-depth b, the variance $\sigma_{0}^{2}$ of the first sampled measurements, and the mean ${\bar{y}}_{0}^{}$ of the first sampled measurements as input variables of the relative PSNR model. In this paper, we designed four neurons in the input layer, one neuron in the output layer, and two layers in the hidden layer for the relative PSNR network, as shown in Figure 2. The mathematical form of the relative PSNR model can be expressed as follows:

(23) $$u_{1}={\left[m,b,\sigma_{0}^{2},{\bar{y}}_{0}\right]}^{T}\;u_{j}=g(W_{j-1}u_{j-1}+d_{j-1})\;,\;2\leq j<4\;F=W_{j-1}u_{j-1}+d_{j-1}\;,\;j=4,$$

where $g(v)\;=\frac{2}{1+e^{-2v}}-1$ is an activation function, $u_{1}$ is the input variable vector, F is the relative PSNR as the output, j is the number of network layers, and $W_{j},d_{j}$ is the model parameter. When training the network, the loss function uses the mean squared error (MSE) between the actual value and the estimated value.

## 5. Rate-Distortion Optimization for Sampling Rate and Bit-Depth

In this part, we use the designed bit-rate model and relative PSNR model to optimize the sampling rate and bit-depth jointly.

### 5.1. Rate-Distortion Optimization Algorithm

We introduced the relative PSNR substitution distortion into problem (5). The optimization problem of sampling rate m and bit-depth b can be expressed as follows:

(24) $$\begin{array}{l} \max F(b,m,x)\\ s.t.\;R\leq R_{\max} \end{array}$$

From bit-rate model (17), let $R=R_{\max}$; there is a correspondence between the sampling rate and the quantization depth as follows:

(25) $$m=\frac{R_{\max}-{c^{\prime }}_{3}}{{c^{\prime }}_{1}b+{c^{\prime }}_{2}{\left(2^{b}-1\right)}^{2}+{c^{\prime }}_{4}\sigma_{0}^{2}+{c^{\prime }}_{5}\Delta_{y_{0}}^{}+\frac{{c^{\prime }}_{6}}{\Delta_{y_{0}}^{2}}+{c^{\prime }}_{7}}.$$

The number of bit-depth b is less than the number of sampling rate m, and is much less than the number of combinations for bit-depth and sampling rate. According to Equation (25), the number of candidate parameters of problem (5) can be reduced to the same number as the bit-depth. 

Therefore, the proposed adaptive CS image coding framework with rate-distortion optimization follows the main steps below:

(1) Input: $R_{\max}$
(2) First sampling.

Sampling rate is $m_{0}$, and the original image is measured to obtain partial measurements $y_{0}\in {\mathbb{R}}^{round(Nm_{0})\times 1}$.

(3) Extracting features.

Calculate the mean ${\bar{y}}_{0}$, the variance $\sigma_{0}^{2}$, the maximum ${y^{\prime }}_{\max}$ and the minimum ${y^{\prime }}_{\min}$ of $y_{0}$.

(4) Reducing the candidate set.

Calculate the sampling rate m corresponding to each bit-depth b based on Equation (25), obtaining a candidate parameter set $\left\{\left(b_{1},m_{1}\right),\ldots ,\left(b_{\lambda },m_{\lambda }\right)\right\}$, where λ represents the number of quantization depths.

(5) Estimating the optimal parameters.

Estimate the relative PSNR of all candidate parameters according to the four-layered feedforward neural network, and select the parameter $\left(b^{*},m^{*}\right)$ for which relative PSNR is best. $m^{*}$ is the optimized sampling rate and $b^{*}$ is the optimized bit-depth.

(6) Second sampling.

Sampling rate is $m=m^{*}-m_{0}$, and the original image is measured to obtain the remaining measurements.

(7) Quantization and entropy coding.

The measurements of the two samplings are quantized using the bit-depth $b^{*}$, and then are entropy encoded. 

### 5.2. Model Parameter Estimation for the Bit-Rate Model and the Relative PSNR Model

In order to estimate the model parameters of the proposed average codeword length model and the relative PSNR model, 100 images in the BSDS500 dataset [35] were randomly selected for training, and the BSD68 dataset [36] was used for testing, each image being cropped to a 256 × 256 size. During training, the quantization bit-depth took eight values in {3, 4, …, 10}, and the sampling rate used 49 values which included 40 values in {0.01, 0.02, …, 0.4} and 9 values in {1/30, 1/35, 1/40, …, 1/80}. Each image collected 392 samples, which included the average codeword length, the relative PSNR, and their affecting factors. A total of 39,200 samples were collected for model training. At the encoder, the same orthogonal Gaussian measurement matrix was first used for block CS sampling, in which the image block size was 32 × 32 (the measurement still obeys the approximate Gaussian distribution [26]), and then uniform quantization and arithmetic coding were performed. At the decoder, arithmetic decoding and inverse quantization were first performed, and then CS reconstruction was performed using a non-local low-rank algorithm (NLR-CS) [23], in which the initial image was reconstructed total variation iterative threshold regularization image reconstruction algorithms (BCS-TVIT) [37]. 

The initial sampling rate $m_{0}$ determines the accuracy of the image features estimated by $\sigma_{0}^{2}$ and ${\bar{y}}_{0}^{}$. The larger it is, the better it is to estimate the bit rate and PSNR accurately. However, if $m_{0}$ is too large, there may be unnecessary measurements and calculations. When a Gaussian random matrix is used, the number of measured values for reconstructing a high-quality signal is at least M=O(Klog(N)) [21], so the best choice of the initial sampling rate m should be O(Klog(N))/N, which is difficult to estimate it accurately. We analyzed the sample data of the training set and found that when the sampling rate was lower than 0.013, the visual quality of all reconstructed images was bad, and the PSNR value did not exceed 15 dB. Therefore, we used $m_{0}=0.013$.

As shown in Table 1, the parameters of our model (16) were obtained by least square fitting with the $L_{y_{Q}}$ in the training set. To quantify the accuracy of the fitting, we also measured the mean squared error (MSE), the Pearson correlation coefficient (PCC), and R-squared ($R^{2}$) [38] between actual $L_{y_{Q}}$ and predicted $L_{y_{Q}}$ in the test set. The closer $R^{2}$ and PCC are to 1, the better the degree of fit of the model.

As can be seen from Table 1, all parameters are non-zero except for the value of ${c^{\prime }}_{6}$, which verifies the mapping relationship between the sampling rate m, bit-depth b, variance $\sigma_{0}^{2}$, interval $\Delta_{y_{0}}^{}$, and average codeword length $L_{y_{Q}}$. ${c^{\prime }}_{6}$ is the coefficient of $\frac{1}{\Delta_{y_{0}}^{2}}$, and the value of $\frac{1}{\Delta_{y_{0}}^{2}}$ is very small. When there is a fifth term ${c^{\prime }}_{5}\Delta_{y_{0}}^{}$, the correlation between $\frac{1}{\Delta_{y_{0}}^{2}}$ and $L_{y_{Q}}$ is very weak. ${c^{\prime }}_{6}=0$ indicates that the influence of $\frac{1}{\Delta_{y_{0}}^{2}}$ can be ignored in model (16).

In Table 2, the R-squared of model (12) reaches 0.9809 and the PCC reaches 0.9904. The R-squared of model (16) reaches 0.9903 and the PCC reaches 0.9952, which is better than the estimation of model (12). The results show that both model (12) and model (16) can describe well the relationship between sampling rate m, bit-depth b, variance $\sigma_{0}^{2}$, mean ${\bar{y}}_{0}$, and the average codeword length $L_{y_{Q}}$, and that model (16) is better than model (12). Moreover, bit-rate model (17) based on model (16) has no logarithmic operation, and can quickly calculate the sampling rate based on the bit-depth b and the $R_{\max}$ to narrow the parameter candidate set, which is more conducive to practical application.

When collecting data about the relative PSNR, we took $b_{1}=3,m_{1}=0.013$, $b_{2}=8,m_{2}=0.4$ for $F_{1}、F_{2}、F_{3}、F_{4}$. We used the “newff” function in MATLAB 2018b software for training PSNR, $F_{1}$, $F_{2}$, $F_{3}$ and $F_{4}$, respectively, where the input and the four-layered feedforward neural network are the same. The training and testing performances are shown in Table 3. 

Table 3 shows that the effect of fitting the PSNR using the same input variables and network structure is the worst, because PSNR is calculated from the difference between the original image and the reconstructed image. In addition to being related to the sampling rate m, quantized bit-depth b, and the variance $\sigma_{0}^{2}$ and average ${\bar{y}}_{0}^{}$ of some measurements, PSNR is also closely related to other factors. Compared with the estimated PSNR, the performance of the estimated $F_{1}$, $F_{2}$, $F_{3}$, and $F_{4}$ is improved. Among them, the effect of estimating $F_{4}$ is the best, which shows that the mapping relationship between sampling rate m, bit-depth b, variance $\sigma_{0}^{2}$, mean ${\bar{y}}_{0}^{}$, and $F_{4}$ is closer than that with $F_{1}$, $F_{2}$, and $F_{3}$. Therefore, we chose $F_{4}$ to evaluate distortion.

### 5.3. Computational Complexity of the Rate-Distortion Optimization Algorithm

The additional computational complexity of the rate-distortion optimization for sampling rate and the bit-depth is mainly derived from feature extraction, rate estimation, and relative PSNR estimation.

The calculation of extracting features is mainly from the $\sigma_{0}^{2}$, ${\bar{y}}_{0}$, ${y^{\prime }}_{\max}$, and ${y^{\prime }}_{\min}$ values. Assuming the image size is I×I and the block size is 32 × 32, the number of measurements obtained by the first sampling is $0.013\times I^{2}$. The calculation of ${\bar{y}}_{0}$ requires $0.013\times I^{2}-1$ additions and one multiplication. The calculation of $\sigma_{0}^{2}$ requires $0.013\times I^{2}\times 2-1$ additions and $0.013\times I^{2}+1$ multiplications. The ${y^{\prime }}_{\max}$ and ${y^{\prime }}_{\min}$ require a total of up to $\left(0.013\times I^{2}-1\right)\times 2$ comparisons. Assuming that a comparison requires two subtractions, a total of $\left(0.013\times I^{2}-1\right)\times 4$ subtractions are required. The first sampling requires $0.013\times I^{2}\times 1023$ additions and $0.013\times I^{2}\times 1024$ multiplications. Assuming the same computational complexity of subtraction and addition, extracting features require a total of $0.078\times I^{2}-6$ additions and $0.013\times I^{2}+2$ multiplications. The extracted feature additionally adds 0.11% multiplication and 0.59% addition compared to the first sampling.

The calculation of the rate estimation process mainly comes from the calculation of Equation (25). Since the bit-depth is a finite discrete value, ${\left(2^{b}-1\right)}^{2}$ can be calculated using a lookup table in the equation. At this point, calculating Equation (25) requires seven additions and seven multiplications. We chose seven bit-depths as candidate values, and then Equation (25) had to calculate a total of 49 additions and 49 multiplications.

The calculation of the relative PSNR estimation process mainly comes from the calculation of the neural network model (23). The network input layer has four neurons, and the output layer has one neuron. The network has two hidden layers, each with six neurons. The number of network parameters is 4 × 6 + 6 + 6 × 6 + 6+6 × 1 + 1 = 79. Networks without activation functions include 4 × 6 + 6 × 6 + 6 × 1 = 66 multiplications and 3 × 6 + 6 + 5 × 6 + 6 + 5 + 1 = 66 additions. The hidden layer uses the sigmoid activation function. It is assumed that the series approximation calculates the exponential power. When the precision is $10^{-7}$, it takes about 60 multiplications and 10 additions to calculate an activation function. Calculating 12 activation functions requires 720 multiplications and 120 additions. The calculation of the network model once is about 782 multiplications and 182 additions. If we select seven bit-depths as candidate values, we must calculate the relative PSNR of seven candidate parameters. In this case, we had to calculate 5474 multiplications and 1274 additions in total.

A measurement requires 1024 multiplications and 1023 additions. The computation of the estimated bit rate and relative PSNR does not exceed the multiplications of six measurements and the additions of two measurements. When compressing an image of size 256 × 256, the first sampling can obtain 852 measurements. The computation of the estimated bit rate and relative PSNR increases the multiplications by 6/852≈0.7% and the additions by 2/852≈0.23%. Compared with the computation of the first sampling, the additional computation of the entire rate-distortion optimization process increases by 0.81% multiplication and 0.82% addition.

## 6. Numerical Results and Analysis

**Figure 3:**
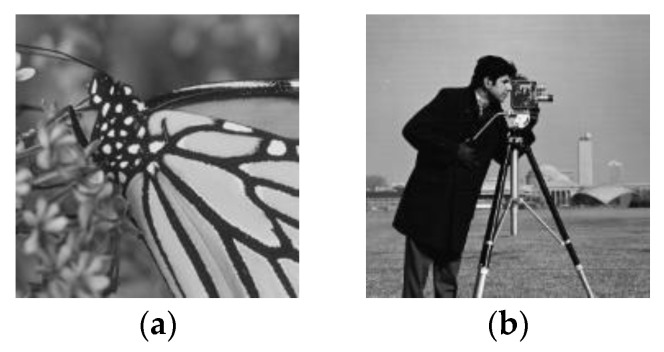

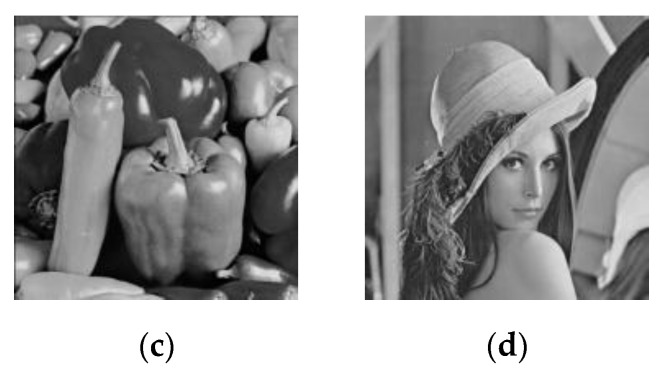
Four testing images. (**a**) Monarch; (**b**) Cameraman; (**c**) Peppers; (**d**) Lena.

We performed some numerical tests to check the performance of the proposed algorithm. In our simulation, we tested Monarch, Cameraman, Peppers, and Lena (as shown in Figure 3), as well as 68 images from the BSD68 dataset, which were cut to a size of 256 × 256. All simulations were run on MATLAB 2018b software on a Core i5 machine with 8 GB of RAM.

In order to verify the accuracy of the bit-rate model, we set the target bit rate to 0.1, 0.2, ..., 1 bit per pixel (bpp), where the bit-depth set was {3, 4, ..., 9}. The actual bit rate of the optimized result with the proposed algorithm is shown in Table 4 and Table 5.

In Table 4, the error represents the difference of the actual bit rate minus the target bit rate, the error percentage represents the percentage of the error in the target bit rate, and the absolute error percentage is the absolute of the error percentage. Table 4 shows that the actual bit rate is very close to the target bit rate for Monarch, Cameraman, Peppers, and Lena coded by the proposed method. When the target bit rate is 0.1, although the bit-rate error percentage is the largest, the error is between 0.0017 bpp and 0.0027 bpp, which belongs to a smaller range.

It can be seen from Table 5 that the average of the actual bit rate is very close to the target bit rate for the BSD68 test set coded by the proposed method. The average absolute error percentage in the BSD68 test set is between 1.81% and 2.33%, which is slightly higher than the results in Table 4. According to specific data, it can be observed that the bit-rate error of image “test20” is the largest in the BSD68 test set. This is due to a large number of white background areas in image “test20”, which leads to multiples of the entropy coder far exceeding other images for quantized measurements. Even so, the compression performance of “test20” is still better than the CS encoding method without the entropy coder.

In order to verify the validity of the relative PSNR model, we first calculated the parameter candidate set $\left\{\left(b_{1},m_{1}\right),\ldots ,\left(b_{num},m_{num}\right)\right\}$ based on Equation (25) for each image in the test set (BSD68), then performed compression decoding on each parameter and calculated the PSNR value of the decoded image, and finally compared the real PSNR and the degree of PSNR based on the relative PSNR model. The results are shown in Table 6.

In Table 6, the optimal percentage indicates the percentage of the number of images in which the relative PSNR model selects the optimal parameters from the candidate set. The suboptimal percentage indicates the percentage of the number of images in which the relative PSNR model selects the suboptimal parameters from the candidate set. The average PSNR error represents the average of the PSNR errors for all test images. When calculating the PSNR error of an image, we first calculated the candidate parameter $\left\{\left(b_{1},m_{1}\right),\ldots ,\left(b_{num},m_{num}\right)\right\}$ based on the target bit rate and Equation (25). Second, we calculated the PSNR of decoded images for all candidate parameters, then estimated the optimal parameters based on the relative PSNR model and found the corresponding PSNR. Finally, we took the absolute difference between the PSNR of the estimated parameters and the maximum PSNR as the PSNR error. Table 6 shows that the percentage of the optimal parameters and the suboptimal parameters is between 92.65% and 100%. When the target bit rate is 1 bpp, the ratio of successful selection of the optimal and suboptimal is at least 88.24%. This occurs because, with the increase in the target bit rate, the PSNR difference between different parameters is small, resulting in estimation errors. Although the optimal percentage is not very high, the average PSNR error is between 0.128 dB and 0.299 dB, which is a small range. There is some error in the optimization result of the relative PSNR model, and it is acceptable compared to the computational complexity of undergoing the distortion cost of all candidate parameters.

**Figure 4:**
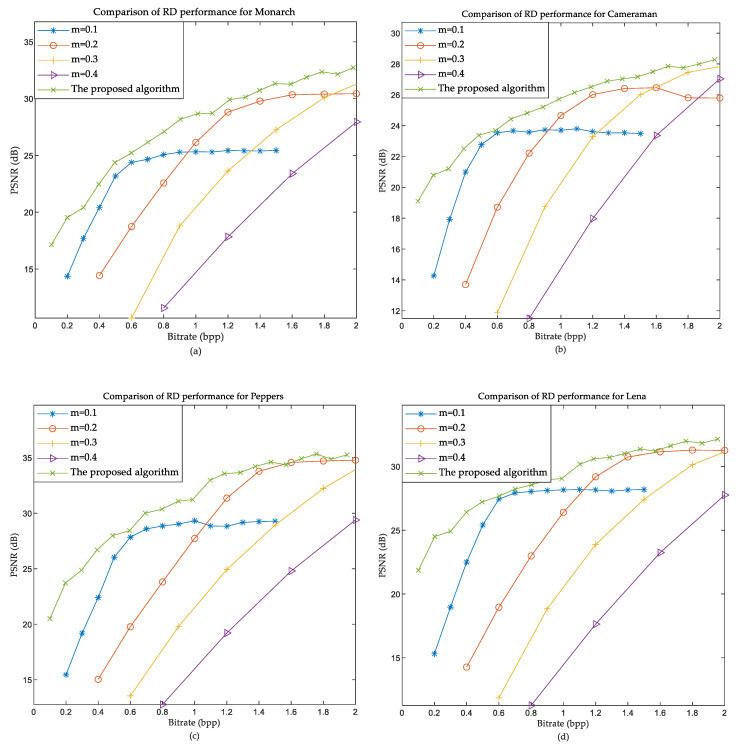
Comparison of rate-distortion (RD) performances. (**a**) Monarch; (**b**) Cameraman; (**c**) Peppers; (**d**) Lena.

In order to verify the rate-distortion (RD) performance of the proposed method, a comparative experiment was performed with the conventional CS coding method. In the traditional method, we first used the fixed m = 0.1 and verified b to obtain different bit rates; we then obtained different RD curves by using m = 0.2, 0.3, and 0.4, respectively. Taking the images Cameraman and Lena as examples, we easily obtained the five different RD curves shown in Figure 4.

As can be seen from Figure 4, the rate-distortion performance of the proposed method is the best. The main reason is that the method of fixed sampling rate cannot adjust the sampling rate. Nevertheless, the proposed method can adaptively select the sampling rate according to the bit-rate model and the bit-depth, and we combined the relative PSNR model for parameter optimization. The proposed method has therefore the best rate-distortion performance.

## 7. Conclusions

Both quantization and CS sampling cause distortion in a CS-based imaging scheme. Given a bit budget, it is essential to assign quantization bit-depth and sampling rate. Rate-distortion optimization plays a crucial role for the image/video encoder. In this work, we proposed a low-complexity rate-distortion optimization method to jointly optimize the sampling rate and the quantization bit-depth through the proposed bit-rate model and distortion model. First, we proposed a simple bit-rate model based on the information entropy and the second-order Taylor expansion. The bit-rate model can estimate the sampling rate according to the quantization bit at a given target bit rate, thereby reducing the range of the parameter candidate set. Second, we introduced the relative PSNR as the equivalent function of distortion. We proposed a four-layered feedforward neural network to learn the relative PSNR model, where the model can improve the accuracy of estimating the level of distortion. The experimental results show that the actual bit rate of compression with the proposed method is very close to the target bit rate. Compared with the traditional CS coding method, this method provides a better rate-distortion performance with very little extra computation.

## Figures and Tables

**Table 1:** Parameters of the average codeword length model (16).

${c^{\prime }}_{1}$	${c^{\prime }}_{2}$	${c^{\prime }}_{3}$	${c^{\prime }}_{4}$	${c^{\prime }}_{5}$	${c^{\prime }}_{6}$	${c^{\prime }}_{7}$
1.06741	1.65688 × 10^−7^	0.012574	4.48157 × 10^−5^	−0.001619	0	−0.769651

**Table 2:** Fitting accuracy of model (12) and model (16).

	$R^{2}$	PCC	MSE
Model (12)	0.9809	0.9904	0.10574
Model (16)	0.9903	0.9952	0.05035

**Table 3:** Fitting accuracy of $F_{1}、F_{2}、F_{3}、F_{4}$

Output	Training Set		Test Set	
	$R^{2}$	PCC	$R^{2}$	PCC
PSNR	0.7014	0.847	0.6994	0.8449
$F_{1}$	0.8174	0.9041	0.8653	0.9302
$F_{2}$	0.9002	0.9488	0.8317	0.9120
$F_{3}$	0.9002	0.9488	0.8302	0.9112
$F_{4}$	0.9385	0.9687	0.9571	0.9783

**Table 4:** Comparison of target bit rate with actual bit rate for Monarch, Cameraman, Peppers, and Lena.

**Image**	**Target Bit Rate**	**0.1**	**0.2**	**0.3**	**0.4**	**0.5**
Monarch	Actual bit-rate	0.1027	0.2020	0.3023	0.3992	0.4990
	error	0.0027	0.0020	0.0023	−0.0008	−0.0010
	Error percentage (%)	2.67	1.01	0.76	−0.19	−0.19
Cameraman	Actual bit-rate	0.1017	0.1990	0.2956	0.3921	0.4872
	error	0.0017	−0.0010	−0.0044	−0.0079	−0.0128
	Error percentage (%)	1.74	−0.50	−1.47	−1.97	−2.56
Peppers	Actual bit-rate	0.1012	0.1996	0.2986	0.3964	0.4937
	error	0.0012	−0.0004	−0.0014	−0.0036	−0.0063
	Error percentage (%)	1.24	−0.19	−0.47	−0.91	−1.26
Lena	Actual bit-rate	0.1018	0.2038	0.3030	0.4015	0.5011
	error	0.0018	0.0038	0.0030	0.0015	0.0011
	Error percentage (%)	1.76	1.90	1.01	0.38	0.22
Average of absolute error percentage (%)		1.85	0.90	0.93	0.86	1.06
**Image**	**Target Bit Rate**	**0.6**	**0.7**	**0.8**	**0.9**	**1**
Monarch	Actual bit-rate	0.6033	0.7054	0.8088	0.9093	1.0169
	error	0.0033	0.0054	0.0088	0.0093	0.0169
	Error percentage (%)	0.55	0.77	1.10	1.03	1.69
Cameraman	Actual bit-rate	0.5944	0.6899	0.7932	0.8931	0.9994
	error	−0.0056	−0.0101	−0.0068	−0.0069	−0.0006
	Error percentage (%)	−0.93	−1.44	−0.86	−0.77	−0.06
Peppers	Actual bit-rate	0.5994	0.6975	0.8037	0.9035	0.9931
	error	−0.0006	−0.0025	0.0037	0.0035	−0.0069
	Error percentage (%)	−0.10	−0.36	0.46	0.39	−0.69
Lena	Actual bit-rate	0.6071	0.7071	0.8085	0.9091	0.9980
	error	0.0071	0.0071	0.0085	0.0091	−0.0020
	Error percentage (%)	1.19	1.02	1.06	1.01	−0.20
Average of absolute error percentage (%)		0.69	0.90	0.87	0.80	0.66

**Table 5:** Comparison of target bit rate with actual bit rate for BSD68 test set.

**Image**	**Target Bit Rate**		**0.1**	**0.2**	**0.3**	**0.4**	**0.5**
BSD68 test set	Actual bit rate	Maximum	0.1039	0.2079	0.3126	0.4151	0.5191
		Minimum	0.0757	0.1653	0.2451	0.3321	0.4158
		Average	0.0997	0.2003	0.3003	0.3965	0.4953
		Average of absolute error percentage (%)	2.33	2.06	1.98	1.88	1.81
**Image**	**Target Bit Rate**		**0.6**	**0.7**	**0.8**	**0.9**	**1**
BSD68 test set	Actual bit rate	Maximum	0.6242	0.7271	0.8352	0.9398	1.0484
		Minimum	0.5104	0.5977	0.6839	0.7682	0.8494
		Average	0.5963	0.6987	0.7986	0.8975	0.9954
		Average of absolute error percentage (%)	1.79	1.84	1.85	1.90	1.92

**Table 6:** Performance of the relative PSNR.

**Target Bit Rate**	**0.1**	**0.2**	**0.3**	**0.4**	**0.5**
Optimal percentage (%)	69.12	45.59	33.82	50.00	48.53
Suboptimal percentage (%)	30.88	47.05	61.77	42.65	45.59
Sum of the above (%)	100.00	92.65	95.59	92.65	94.12
Average PSNR error (dB)	0.174	0.134	0.226	0.128	0.146
**Target Bit Rate**	**0.6**	**0.7**	**0.8**	**0.9**	**1**
Optimal percentage (%)	42.65	50.00	47.06	57.35	45.59
Suboptimal percentage (%)	51.47	45.59	48.53	35.29	42.65
Sum of the above (%)	94.12	95.59	95.59	92.65	88.24
Average PSNR error (dB)	0.216	0.184	0.212	0.204	0.299

## Appendix A

The logarithmic function $\log_{2}(a\prod_{i=1}^{n}x_{i}+C)$ has a second-order approximate expression function and is given as follows:

$$\log_{2}(a\prod_{i=1}^{n}x_{i}+C)\approx \sum_{i=1}^{n}\log_{2}(x_{i})+\sum_{i=1}^{n}c_{i}x_{i}+c_{n+1}$$

**Proof.** Let

$$g_{1}(x_{1},x_{2},\ldots ,x_{n})=\log_{2}(a\prod_{i=1}^{n}x_{i}+C),\;g_{2}(x_{1},x_{2},\ldots ,x_{n})=\log_{2}(a\prod_{i=1}^{n}x_{i}+C)$$

when n = 1, $g_{1}(x_{1})=\log_{2}(ax_{1}+C)$, $g_{2}(x_{1})=\log_{2}(ax_{1})$. It is easy to find two points $x_{1}^{\left(1\right)}$ and $x_{1}^{\left(2\right)}$, which satisfy $ax_{1}^{\left(1\right)}+C=ax_{1}^{\left(2\right)}$. Using Taylor’s second-order expansion formula, we obtain the following:

$$g_{1}(x_{1})=g_{1}(x_{1}^{\left(1\right)})+\frac{\partial g_{1}(x_{1}^{\left(1\right)})}{\partial x_{1}}(x_{1}-x_{1}^{\left(1\right)})+\frac{\partial^{2}g_{1}(x_{1}^{\left(1\right)})}{\partial x_{1}\partial x_{1}}{\left(x_{1}-x_{1}^{\left(1\right)}\right)}^{2}$$

$$g_{2}(x_{1})=g_{1}(x_{1}^{\left(2\right)})+\frac{\partial g_{1}(x_{1}^{\left(2\right)})}{\partial x_{1}}(x_{1}-x_{1}^{\left(2\right)})+\frac{\partial^{2}g_{1}(x_{1}^{\left(2\right)})}{\partial x_{1}\partial x_{1}}{\left(x_{1}-x_{1}^{\left(2\right)}\right)}^{2}$$

According to $ax_{1}^{\left(1\right)}+C=ax_{1}^{\left(2\right)}$, we obtain the following:

$$g_{1}(x_{1}^{\left(1\right)})=g_{1}(x_{1}^{\left(2\right)}),\frac{\partial g_{1}(x_{1}^{\left(1\right)})}{\partial x_{1}}=\frac{\partial g_{1}(x_{1}^{\left(2\right)})}{\partial x_{1}},\frac{\partial^{2}g_{1}(x_{1}^{\left(1\right)})}{\partial x_{1}\partial x_{1}}=\frac{\partial^{2}g_{1}(x_{1}^{\left(2\right)})}{\partial x_{1}\partial x_{1}}$$

so,

$$g_{1}(x_{1})-g_{2}(x_{1})=\frac{\partial g_{1}(x_{1}^{\left(1\right)})}{\partial x_{1}}(x_{1}^{\left(2\right)}-x_{1}^{\left(1\right)})+\frac{\partial^{2}g_{1}(x_{1}^{\left(1\right)})}{\partial x_{1}\partial x_{1}}(2x_{1}^{\left(2\right)}-2x_{1}^{\left(1\right)})x_{1}+\frac{\partial^{2}g_{1}(x_{1}^{\left(1\right)})}{\partial x_{1}\partial x_{1}}\left({\left(x_{1}^{\left(1\right)}\right)}^{2}-{\left(x_{1}^{\left(2\right)}\right)}^{2}\right).$$

Therefore,

$$\log_{2}(ax_{1}+C)\approx \log_{2}(ax_{1})+c_{1}x_{1}+c_{2}$$

where

$$c_{1}=\frac{\partial^{2}g_{1}(x_{1}^{\left(1\right)})}{\partial x_{1}\partial x_{1}}(2x_{1}^{\left(2\right)}-2x_{1}^{\left(1\right)})=\frac{-a^{2}(2x_{1}^{\left(2\right)}-2x_{1}^{\left(1\right)})}{{\left(ax_{1}^{\left(1\right)}+C\right)}^{2}}$$

and

$$c_{2}=\frac{\partial g_{1}(x_{1}^{\left(1\right)})}{\partial x_{1}}(x_{1}^{\left(2\right)}-x_{1}^{\left(1\right)})+\frac{\partial^{2}g_{1}(x_{1}^{\left(1\right)})}{\partial x_{1}\partial x_{1}}\left({\left(x_{1}^{\left(1\right)}\right)}^{2}-{\left(x_{1}^{\left(2\right)}\right)}^{2}\right)=\frac{a\left(x_{1}^{\left(2\right)}-x_{1}^{\left(1\right)}\right)}{\left(ax_{1}^{\left(1\right)}+C\right)}-\frac{a^{2}\left({\left(x_{1}^{\left(1\right)}\right)}^{2}-{\left(x_{1}^{\left(2\right)}\right)}^{2}\right)}{{\left(ax_{1}^{\left(1\right)}+C\right)}^{2}}.$$

when n = 2, $g_{1}(x_{1},x_{2})=\log_{2}(ax_{1}x_{2}+C)$, $g_{2}(x_{1},x_{2})=\log_{2}(ax_{1}x_{2})$. We can also find two points $\left(x_{1}^{\left(1\right)},x_{2}^{\left(1\right)}\right)$ and $\left(x_{1}^{\left(2\right)},x_{2}^{\left(2\right)}\right)$, which satisfy

$$ax_{1}^{\left(1\right)}x_{2}^{\left(1\right)}+C=ax_{1}^{\left(2\right)}x_{2}^{\left(2\right)}$$

Using Taylor’s second-order expansion formula, we obtain the following:

$$\begin{array}{cc} g_{1}(x_{1},x_{2})= & g_{1}(x_{1}^{\left(1\right)},x_{2}^{\left(1\right)})+\frac{\partial g_{1}(x_{1}^{\left(1\right)},x_{2}^{\left(1\right)})}{\partial x_{1}}(x_{1}-x_{1}^{\left(1\right)})+\frac{\partial g_{1}(x_{1}^{\left(1\right)},x_{2}^{\left(1\right)})}{\partial x_{2}}(x_{2}-x_{2}^{\left(1\right)})\\ & +\frac{\partial^{2}g_{1}(x_{1}^{\left(1\right)},x_{2}^{\left(1\right)})}{2\partial x_{1}\partial x_{1}}{\left(x_{1}-x_{1}^{\left(1\right)}\right)}^{2}+\frac{\partial^{2}g_{1}(x_{1}^{\left(1\right)},x_{2}^{\left(1\right)})}{2\partial x_{2}\partial x_{2}}{\left(x_{2}-x_{2}^{\left(1\right)}\right)}^{2}\\ & +\frac{\partial^{2}g_{1}(x_{1}^{\left(1\right)},x_{2}^{\left(1\right)})}{\partial x_{1}\partial x_{2}}(x_{1}-x_{1}^{\left(1\right)})(x_{2}-x_{2}^{\left(1\right)}) \end{array}$$

$$\begin{array}{cc} g_{2}(x_{1},x_{2})= & g_{2}(x_{1}^{\left(2\right)},x_{2}^{\left(2\right)})+\frac{\partial g_{2}(x_{1}^{\left(2\right)},x_{2}^{\left(2\right)})}{\partial x_{1}}(x_{1}-x_{1}^{\left(2\right)})+\frac{\partial g_{2}(x_{1}^{\left(2\right)},x_{2}^{\left(2\right)})}{\partial x_{2}}(x_{2}-x_{2}^{\left(2\right)})\\ & +\frac{\partial^{2}g_{2}(x_{1}^{\left(2\right)},x_{2}^{\left(2\right)})}{2\partial x_{1}\partial x_{1}}{\left(x_{1}-x_{1}^{\left(2\right)}\right)}^{2}+\frac{\partial^{2}g_{2}(x_{1}^{\left(2\right)},x_{2}^{\left(2\right)})}{2\partial x_{2}\partial x_{2}}{\left(x_{2}-x_{2}^{\left(2\right)}\right)}^{2}\\ & +\frac{\partial^{2}g_{2}(x_{1}^{\left(2\right)},x_{2}^{\left(2\right)})}{\partial x_{1}\partial x_{2}}(x_{1}-x_{1}^{\left(2\right)})(x_{2}-x_{2}^{\left(2\right)}) \end{array}$$

The k-order partial derivative of $g_{1}(x_{1}^{},x_{2}^{})$ at point $\left(x_{1}^{\left(1\right)},x_{2}^{\left(1\right)}\right)$ are equal to the k-order partial derivative of $g_{2}(x_{1}^{},x_{2}^{})$ at point $\left(x_{1}^{\left(2\right)},x_{2}^{\left(2\right)}\right)$ based on $ax_{1}^{\left(1\right)}x_{2}^{\left(1\right)}+C=ax_{1}^{\left(2\right)}x_{2}^{\left(2\right)}$ and $g_{1}(x_{1}^{\left(1\right)},x_{2}^{\left(1\right)})=g_{2}(x_{1}^{\left(2\right)},x_{2}^{\left(2\right)})$, where k=1,…,n. So

$$\begin{array}{cc} g_{1}(x_{1},x_{2})- & g_{2}(x_{1},x_{2})=\frac{\partial g_{1}(x_{1}^{\left(1\right)},x_{2}^{\left(1\right)})}{\partial x_{1}}(x_{1}^{\left(2\right)}-x_{1}^{\left(1\right)})+\frac{\partial g_{1}(x_{1}^{\left(1\right)},x_{2}^{\left(1\right)})}{\partial x_{2}}(x_{2}^{\left(2\right)}-x_{2}^{\left(1\right)})\\ & +\frac{\partial^{2}g_{1}(x_{1}^{\left(1\right)},x_{2}^{\left(1\right)})}{2\partial x_{1}\partial x_{1}}\left[2(x_{1}^{\left(2\right)}-x_{1}^{\left(1\right)})x_{1}+{\left(x_{1}^{\left(1\right)}\right)}^{2}-{\left(x_{1}^{\left(2\right)}\right)}^{2}\right]\\ & +\frac{\partial^{2}g_{1}(x_{1}^{\left(1\right)},x_{2}^{\left(1\right)})}{2\partial x_{2}\partial x_{2}}\left[2(x_{2}^{\left(2\right)}-x_{2}^{\left(1\right)})x_{2}+{\left(x_{2}^{\left(1\right)}\right)}^{2}-{\left(x_{2}^{\left(2\right)}\right)}^{2}\right]\\ & +\frac{\partial^{2}g_{1}(x_{1}^{\left(1\right)},x_{2}^{\left(1\right)})}{\partial x_{1}\partial x_{2}}\left[(x_{2}^{\left(2\right)}-x_{2}^{\left(1\right)})x_{1}+(x_{1}^{\left(2\right)}-x_{1}^{\left(1\right)})x_{2}+x_{1}^{\left(1\right)}x_{2}^{\left(1\right)}-x_{1}^{\left(2\right)}x_{2}^{\left(2\right)}\right] \end{array}$$

Therefore, $\log_{2}(ax_{1}x_{2}+C)\approx \log_{2}(ax_{1}x_{2})+\sum_{i=1}^{2}c_{i}x_{i}+c_{3}$, where

$$c_{1}=\frac{\partial^{2}g_{1}(x_{1}^{\left(1\right)},x_{2}^{\left(1\right)})}{\partial x_{1}\partial x_{1}}(x_{1}^{\left(2\right)}-x_{1}^{\left(1\right)})+\frac{\partial^{2}g_{1}(x_{1}^{\left(1\right)},x_{2}^{\left(1\right)})}{2\partial x_{1}\partial x_{2}}(x_{2}^{\left(2\right)}-x_{2}^{\left(1\right)})$$

$$c_{2}=\frac{\partial^{2}g_{1}(x_{1}^{\left(1\right)},x_{2}^{\left(1\right)})}{\partial x_{2}\partial x_{2}}(x_{2}^{\left(2\right)}-x_{2}^{\left(1\right)})+\frac{\partial^{2}g_{1}(x_{1}^{\left(1\right)},x_{2}^{\left(1\right)})}{2\partial x_{1}\partial x_{2}}(x_{1}^{\left(2\right)}-x_{1}^{\left(1\right)})$$

$$\begin{array}{c} c_{3}=\frac{\partial g_{1}(x_{1}^{\left(1\right)},x_{2}^{\left(1\right)})}{\partial x_{1}}(x_{1}^{\left(2\right)}-x_{1}^{\left(1\right)})+\frac{\partial g_{1}(x_{1}^{\left(1\right)},x_{2}^{\left(1\right)})}{\partial x_{2}}(x_{2}^{\left(2\right)}-x_{2}^{\left(1\right)})+\frac{\partial^{2}g_{1}(x_{1}^{\left(1\right)},x_{2}^{\left(1\right)})}{2\partial x_{1}\partial x_{1}}\left[{\left(x_{1}^{\left(1\right)}\right)}^{2}-{\left(x_{1}^{\left(2\right)}\right)}^{2}\right]\\ +\frac{\partial^{2}g_{1}(x_{1}^{\left(1\right)},x_{2}^{\left(1\right)})}{2\partial x_{2}\partial x_{2}}\left[{\left(x_{2}^{\left(1\right)}\right)}^{2}-{\left(x_{2}^{\left(2\right)}\right)}^{2}\right]\;+\frac{\partial^{2}g_{1}(x_{1}^{\left(1\right)},x_{2}^{\left(1\right)})}{\partial x_{1}\partial x_{2}}\left(x_{1}^{\left(1\right)}x_{2}^{\left(1\right)}-x_{1}^{\left(2\right)}x_{2}^{\left(2\right)}\right) \end{array}$$

In the same way, we only need to find two sets of points that satisfy $a\prod_{i=1}^{n}x_{i}+C=a\prod_{i=1}^{n}x_{i}$, and use the second-order Taylor series expansion to obtain the following:

$$\log_{2}(a\prod_{i=1}^{n}x_{i}+C)\approx \log_{2}(a\prod_{i=1}^{n}x_{i})+\sum_{i=1}^{n}c_{i}x_{i}+c_{n+1}$$

According to $\log_{2}(a\prod_{i=1}^{n}x_{i})=\sum_{i=1}^{n}\log_{2}(x_{i})+\log_{2}(a)$, we can obtain the following:

$$\log_{2}(a\prod_{i=1}^{n}x_{i}+C)\approx \sum_{i=1}^{n}\log_{2}(x_{i})+\sum_{i=1}^{n}c_{i}x_{i}+c_{n+2}$$

where $c_{n+2}=c_{n+1}+\log_{2}(a)$. □
